# Understanding crown shyness from a 3-D perspective

**DOI:** 10.1093/aob/mcab035

**Published:** 2021-03-13

**Authors:** Jens van der Zee, Alvaro Lau, Alexander Shenkin

**Affiliations:** 1 Laboratory of Geo-Information Science and Remote Sensing, Wageningen University & Research, Wageningen, the Netherlands; 2 Environmental Change Institute, School of Geography and the Environment, University of Oxford, Oxford, UK

**Keywords:** Crown shyness, complementarity, shapes, LiDAR, canopy, tree slenderness, 3-D, tree morphology

## Abstract

**Background and Aims:**

Crown shyness describes the phenomenon whereby tree crowns avoid growing into each other, producing a puzzle-like pattern of complementary tree crowns in the canopy. Previous studies found that tree slenderness plays a role in the development of crown shyness. Attempts to quantify crown shyness have largely been confined to 2-D approaches. This study aimed to expand the current set of metrics for crown shyness by quantifying the characteristic of 3-D surface complementarity between trees displaying crown shyness, using LiDAR-derived tree point clouds. Subsequently, the relationship between crown surface complementarity and slenderness of trees was assessed.

**Methods:**

Fourteen trees were scanned using a laser scanning device. Individual tree points clouds were extracted semi-automatically and manually corrected where needed. A metric that quantifies the surface complementarity (*S*_c_) of a pair of protein molecules is applied to point clouds of pairs of adjacent trees. Then 3-D tree crown surfaces were generated from point clouds by computing their α shapes.

**Key Results:**

Tree pairs that were visually determined to have overlapping crowns scored significantly lower *S*_c_ values than pairs that did not overlap (*n* = 14, *P *< 0.01). Furthermore, average slenderness of pairs of trees correlated positively with their *S*_c_ score (*R*^2^ = 0.484, *P *< 0.01), showing agreement with previous studies on crown shyness.

**Conclusions:**

The characteristic of crown surface complementarity present in trees displaying crown shyness was succesfully quantified using a 3-D surface complementarity metric adopted from molecular biology. Crown surface complementarity showed a positive relationship to tree slenderness, similar to other metrics used for measuring crown shyness. The 3-D metric developed in this study revealed how trees adapt the shape of their crowns to those of adjacent trees and how this is linked to the slenderness of the trees.

## INTRODUCTION

### Context and background

Forest structure can be defined as the spatial arrangement of above-ground biomass in a forest (Von Gadow and Hui, 2002). Examples of forest structural characteristics include the proportions of different size classes, the number of layers in the canopy and the spacing between trees (Aguirre *et al.*, 2003; West *et al.*, 2009; Bohlman and Pacala, 2012). Forest structure plays a key role in many ecological processes and determines to a large degree the functioning of a forest ecosystem. For example, previous studies have shown that forest structure influences primary productivity as it determines the way in which forests capture sunlight (Ishii *et al.*, 2004; Hardiman *et al.*, 2011, 2013; Williams *et al.*, 2017). Moreover, forest structure affects animal and plant communities by the way in which it shapes habitats in the forest (Franklin and Whitelam, 2005; Burrascano *et al.*, 2008; Halpern and Spies, 2008). Additionally, forest structure may regulate a forest’s resilience against disturbances such as windthrow (Ryan, 2002) and fire (Everham and Brokaw, 1996).

Interactions between tree crowns influence forest structure by changing the way trees grow (Muth and Bazzaz, 2003). The vertical and horizontal distributions of branch and foliage material are strongly influenced by the competition for canopy space between adjacent trees (Getzin *et al.*, 2006; Rouvinen and Kuuluvainen, 2011). Competition can lead to trees growing asymmetrical crowns instead of their ‘ideal’ symmetrical shape. This sometimes results in tree crowns showing a degree of ‘crown shyness’, a phenomenon in which tree crowns avoid full canopy closure by leaving small channel-like gaps between their crowns. Previous studies suggest that physical contact between trees plays an important role in constituting crown shyness. For example, abrasion of the outer twigs can create lasting gaps between tree crowns (Putz *et al.*, 1984; Meng *et al.*, 2006; Hajek *et al.*, 2015), but even non-destructive contact can lead trees to direct branch growth away from adjacent tree crowns (Jaffe *et al.*, 1985). It has also been demonstrated that these effects are more pronounced among trees that have slender stems, i.e. a large height to diameter ratio, because they sway more in windy conditions and are thus more likely to collide with neighbouring trees (Rudnicki *et al.*, 2003; Fish *et al.*, 2006; Goudie *et al.*, 2009).

**Fig. 1.**
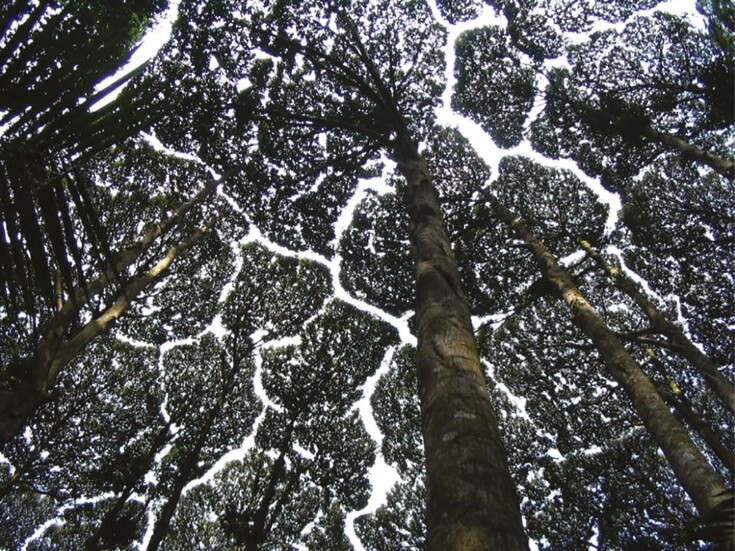
A group of camphor trees (*Dryobalanobs aromatica*) in Malaysia displaying crown shyness. The flat canopy reveals the puzzle-like structure of complementary tree crowns. Image from Wikimedia Commons (2020).

Adaptations of the crown shape resulting from crown collisions can lead to reduced canopy cover and larger distances between tree crowns (Putz *et al.*, 1984; Ezenwenyi and Chukwu, 2017). Moreover, the shapes of neighbouring tree crowns tend to become more complementary, creating the impressive puzzle-like pattern in the canopy termed ‘crown shyness’ (see Fig. 1). These changes in canopy structure can be measured to identify crown shyness. Previous attempts to quantify crown shyness were largely confined to measuring canopy cover or the distance between tree crowns (Putz *et al.*, 1984; Meng *et al.*, 2006). While these metrics are important characteristics of crown shyness, methods for quantifying the feature of crown complementarity are lacking. Filling this gap by developing a metric that quantifies how the crown surfaces of adjacent trees complement each other may improve our understanding of crown shyness.

Surface complementarity modelling is popular in molecular biology where the complementarity of protein molecule surfaces plays an important role in protein aggregation (Li *et al.*, 2013). Molecule surfaces are modeled using α shapes, a general case of the convex hull that allows the presence of both concave and convex sections (Zhou and Yan, 2014). Many molecule surface complementarity estimators quantify complementarity by assessing the degree to which concave and convex sections of a pair of 3-D molecule surfaces coincide (Lawrence and Colman, 1993; Norel *et al.*, 1994). This analysis can be applied to trees; however, this requires the availability of detailed 3-D representations of trees.

The introduction of terrestrial LiDAR in forest settings is revolutionizing forest research (Malhi *et al.*, 2018). The potential of 3-D models has attracted the interest of a wide range of forest research domains, from metabolic scaling in trees (Lau *et al.*, 2018) to estimating forest biomass (Gonzalez de Tanago *et al.*, 2018). The detailed point cloud models of trees derived from terrestrial LiDAR also enable the surface complementarity analysis used in molecular biology to be utilized.

This study aims to expand the set of metrics used to describe crown shyness by measuring the 3-D surface complementarity of adjacent tree crowns using tree point clouds. Subsequently, the relationship between the slenderness of trees and crown surface complementarity is analysed to assess whether there is agreement between the novel 3-D metric for crown shyness and previously developed metrics.

## MATERIALS AND METHODS

### Study site

Terrestrial LiDAR sampling of trees was carried out in January and February 2017 during a field campaign in Guyana for a case study on improving allometric equations for biomass estimation (Lau *et al.*, 2019). The study site was a newly granted logging concession located in the East Berbice region of Guyana (4.48 to 4.56 latitude and –58.22 to –58.15 longitude). The area is covered by dense wet forest and has seen little anthropogenic influence prior to the arrival of the logging company.

### Data

#### LiDAR data.

**Fig. 2.**
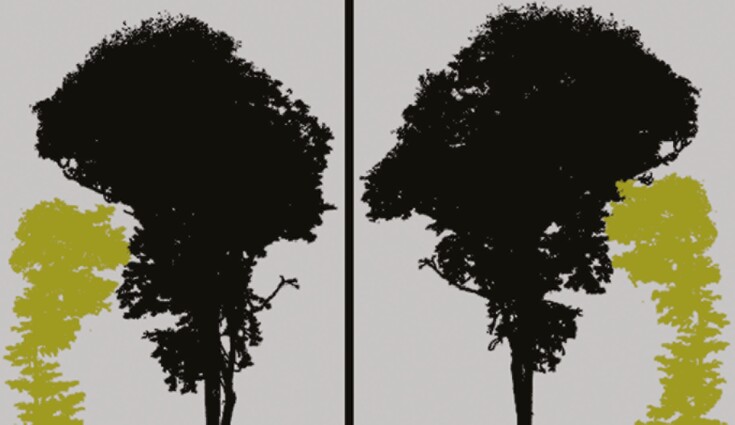
Point clouds of two neighbouring trees viewed from different angles (pair 7 in Table 2).

Over the course of 4 weeks, a total of 106 trees were scanned with a Riegl VZ-400 (Horn, Austria) scanning device using an angular resolution of 0.04°. The trees were scanned from multiple positions in a double circular pattern consisting of an inner ring at 6–8 m and an outer ring at 11–14 m from the focal tree. In total, 14 pairs among 14 individuals were positioned close enough to be considered a pair, with interaction between their crowns (an example is shown in Fig. 2). This was based on a visual inspection of the proximity of the two tree crowns. The individual point clouds of the trees were semi-automatically segmented and manually corrected where needed. For a detailed description. see Lau *et al.* (2019).

#### Tree slenderness.

The relationship between crown surface complementarity and tree slenderness of a pair of trees was tested. Tree height and tree diameter at breast height (DBH) were measured for every tree (see Table 1). Tree slenderness of a pair of trees was calculated as the average slenderness of the two trees:

**Table 1.** Diameter at breast height (DBH), height and species of the trees making up the pairs described in Table 2.

Tree ID	DBH (cm)	Height (m)	Slenderness	Species
40_06	47.5	33.2	0.67	*Licania hypoleuca*
40_10	30.7	26.6	0.87	*Ocotea puberula*
40_11	36.5	23.8	0.65	*Jacaranda copaia*
40_12	36.5	27.0	0.74	*Eperua falcata*
40_24	35.1	28.8	0.82	*Licania guianensis*
40_25	34.4	28.2	0.82	*Aspidosperma excelsum*
60_06	57.0	35.7	0.63	*Emmotum fagifolium*
60_08	52.5	32.6	0.62	*Siparuna surinamensis*
60_12	52.1	30.0	0.58	*Eperua falcata*
60_20	52.7	25.6	0.49	*Chlorocardium rodiei*
60_21	58.0	30.4	0.52	*Emmotum fagifolium*
60_22	59.5	31.4	0.53	*Chlorocardium rodiei*
80_05	82.0	32.0	0.39	*Eperua falcata*
80_21	80.0	37.8	0.47	*Mora gonggrijpii*
80_22	75.0	28.7	0.38	*Chlorocardium rodiei*
100_05	102.0	48.0	0.47	*Bombax globosum*


$$\begin{array}{l} \begin{array}{l} P_{s}=\;\frac{\frac{H_{1}}{D_{1}}+\;\frac{H_{2}}{D_{2}}}{2} \end{array} \end{array}$$ (1)

where *P*_s_ is the slenderness of a pair of trees, *D*_1_ and *D*_2_ are the diameters at breast height and *H*_1_ and *H*_2_ the heights of two adjacent trees. The results are listed in Table 2.

**Table 2.** Crown surface complementarity (*S*_c_) and average slenderness of pairs of trees

Pair #	Tree 1	Tree 2	S_c_	Pair slenderness
1	40_24	60_20	0.70	0.65
2	60_22	80_21	0.39	0.50
3	60_22	60_21	0.13	0.53
4	80_22	60_21	0.26	0.45
5	60_20	60_21	0.47	0.50
6	60_20	80_22	0.15	0.43
7	40_25	80_22	0.63	0.60
8	60_06	100_05	0.70	0.55
9	40_06	100_05	0.75	0.58
10	40_10	60_08	0.67	0.74
11	40_11	60_08	0.40	0.64
12	40_10	40_11	0.68	0.76
13	40_12	60_12	0.65	0.66
14	80_05	60_12	0.20	0.48

See Table 1 for individual tree data.

### Measuring surface complementarity

#### Theoretical background.

 A method for quantifying the complementarity of molecule surfaces is adopted and applied to the point clouds of pairs of trees. The method is adopted from Lawrence and Colman (1993) who computed pair-wise shape complementarity (from here on also referred to as *S*_c_). Lawrence and Colman developed this method for predicting the formation of protein complexes, and it is still being used in ongoing research on protein complex formation (Trachman *et al.*, 2019; Berk *et al.*, 2020).

**Fig. 3.**
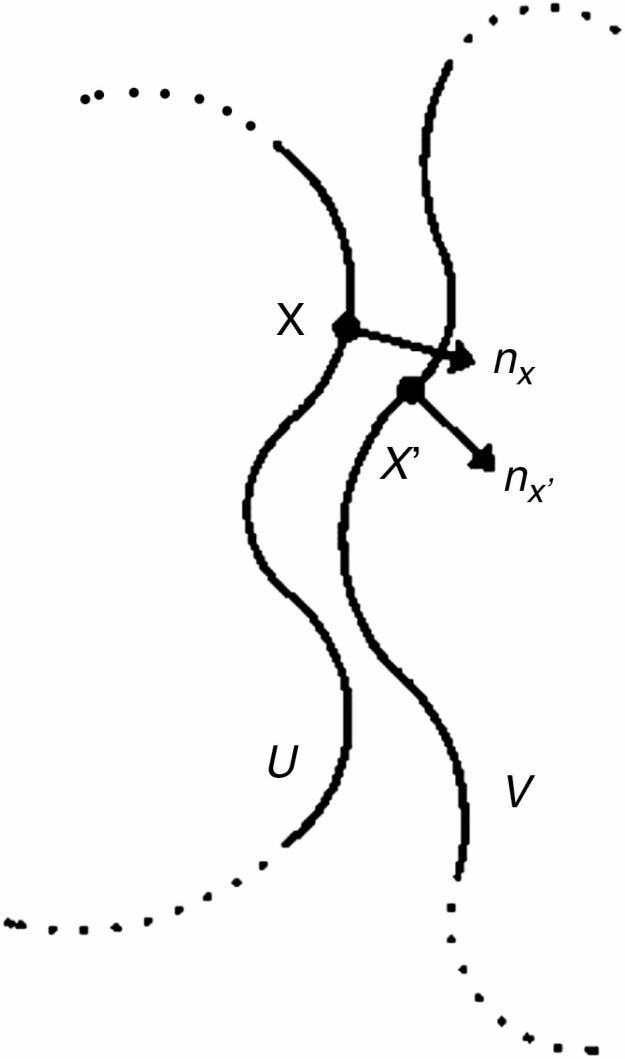
Explanatory schematic drawing of the pairwise complementarity computation, adapted from Lawrence and Colman (1993) with permission.


Figure 3 illustrates the computation of *S*_c_. *U* and *V* (bold continuous lines) are the adjacent parts of the surfaces of two interacting protein molecules (or crown surfaces, in the context of this study). *X* is a point on surface *U* with *n*_*x*_ as its unit normal vector in the direction of surface *V*. *x′* is the point on surface *V* closest to *x* with *n*_*x*_′ as its inward-directed unit normal vector. For every point *x* on surface *U*, the function *S*(*x*, *U*, *V*) is defined as the dot product of *n*_x_ and *n*_*x*_′:

$$\begin{array}{l} \begin{array}{l} S(x,U,V)=n_{x}\cdot {n^{\prime }}_{x} \end{array} \end{array}$$ (2)

The same can be done in the opposite direction, taking the dot product of the normal vector at *x* on surface *V* and the inward normal vector at *x*′ on surface *U*:


$$\begin{array}{l} \begin{array}{l} S\left(x,V,U\right)=n_{x}\cdot {n^{\prime }}_{x} \end{array} \end{array}$$ (3)

*S*(*x*, *U*, *V*) and *S*(*x*, *V*, *U*) can be sampled at points *j* and *k*, respectively. *S*_c_ is then defined as the average of the arithmetic means of the sampled *S*(*x*, *U*, *V*) and *S*(*x*, *V*, *U*) values:


$$\begin{array}{l} \;\begin{array}{l} S_{c}=\;\frac{\frac{1}{j}\overset{\;j}{\underset{i=1}{\sum }}S\left(x,\;U,\;V\right)\;+\;\frac{1}{k}\overset{k}{\underset{i=1}{\sum }}S\left(x,\;V,\;U\right)\;}{2} \end{array} \end{array}$$ (4)

**Fig. 4.**
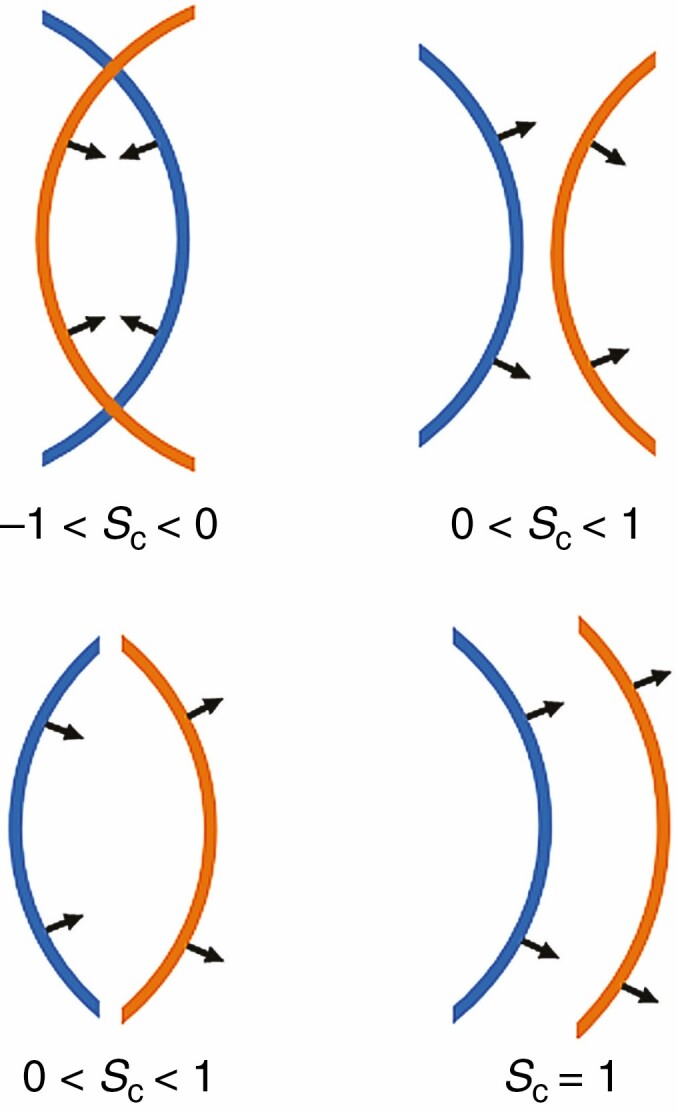
Different arrangements of convex and concave sections with their corresponding *S*_c_ values. Situation A depicts two overlapping surface sections. In situations B and C, both surface sections are convex or concave, respectively. In situation D, a concave section perfectly coincides with a concave section.

In short, *S*_c_ is an average of unit normal vector dot products. The dot product of two unit normal vectors can be interpreted as an expression of how similar the directions of the unit normal vectors are. The value of a unit normal vector dot product ranges from –1 (completely opposed direction of the vectors) to 1 (vector directions line up perfectly). As shown in Fig. 4, the value of dot products between the unit normal vectors of two nearest neighbours on a pair of surfaces is related to how convex and concave sections of the surfaces are arranged. The dot product returns negative values when the surfaces overlap (Fig. 4A). When convex sections on one surface coincide with convex sections on the other surface, the values of the unit normal vector dot product range from 0 to 1 (Fig. 4B). The same holds for coincidence of concave sections on both surfaces (Fig. 4C). The dot product values equal 1 when convex sections on one surface perfectly coincide with concave sections on the other (Fig. 4D).

By applying this analysis to tree crown surfaces, a numerical value for tree crown surface complementarity can be produced. A pair of trees with overlapping tree crown surfaces will score low as a result of the negative values on the overlapping parts. A pair with non-overlapping tree crowns (i.e. a pair that shows crown shyness) scores higher, especially so when concave and convex sections coincide.

#### Segmentation of the interaction zone.

The functions *S*(*x*, *U*, *V*) [eqn (2)] and *S*(*x*, *V*, *U*) [eqn (3)] are evaluated at points on those parts of the surfaces that are interacting (*U* and *V* in Fig. 3). To improve reproducibility and allow the computation of complementarity values for a large number of pairs, a procedure for the automatic segmentation of the surfaces into interacting and non-interacting parts was developed.

**Fig. 5.**
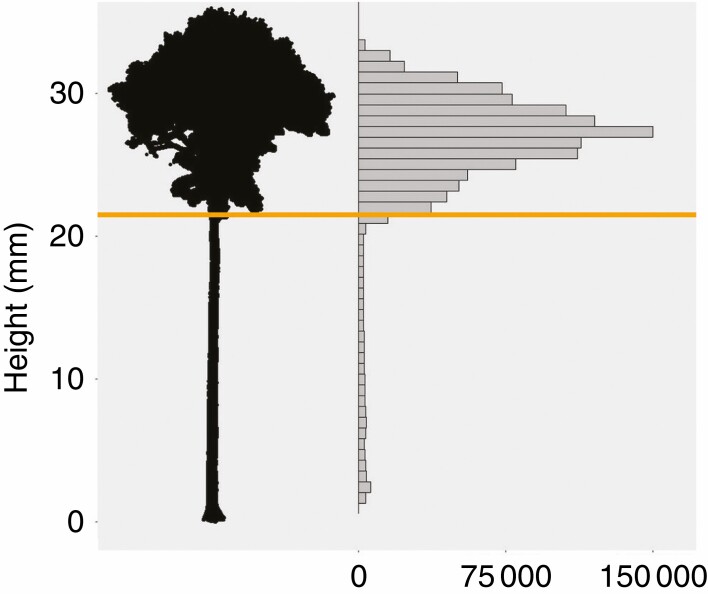
A height projection of a voxelized tree point cloud (left) and its point count histogram of height values (right). The orange line indicates the height value associated with the first bin to exceed the threshold density value (*P* = 0.015 produced satisfactory results for all pairs). This threshold is used to delimit the crown area.

The first step in the procedure is the separation between the bole and the crown of the trees. At this moment, the point clouds are partitioned into voxels (i.e. cubes of a specific size that fill the entire space; Lecigne *et al.*, 2018). The voxels are used to make a vertical profile of the tree point clouds (Fig. 5). The number of points strongly increases in the vertical direction from the first branching point onward. The first histogram bin in the vertical direction with a density value larger than a certain threshold marks the bottom of the tree crown. All points with a height value larger than or equal to the height value associated with that bin are selected and classified as tree crown. Based on a visual assessment, a density threshold of *P* = 0.015, which produced satisfactory results for all pairs in this dataset, was selected.

A two-way nearest-neighbour search in 3-D space is performed between the entire point sets of the two tree crowns, producing a set of points that demarcates the parts of the crowns that are close to each other. The number of points in this set depends on the shape and size of the crowns. Around this set of neighbouring points, a bounding box is created. The bounding box is oriented along the line connecting the centroids of the *x* and *y* co-ordinates of the two entire tree crown point sets. The height of the box is defined by the minimum and maximum height of the points in the nearest-neighbour search result. Points of each crown that are inside this bounding box are selected for surface generation.

#### Surface generation using α shapes.

Tree crown surfaces can be generated using α shapes (Edelsbrunner and Mucke, 1994). The α shape defines the shape of a point set by carving out empty space between the points using a sphere with radius α. In the context of this study, the value of α is always expressed in metres. The α shape algorithm produces a set of boundary points which can be used as input for a Delaunay triangulation to create a surface around the original point set. When α = ∞, the α shape of a point set is identical to the convex hull of that point set. Smaller α values allow cavities to be present in the shape. This property allows the characterization of both convex and concave sections on the surfaces of the tree crowns. The effect of α value selection on the computation of *S*_c_ is evaluated by assessing the results of using a range of α values.

The α shape computation is performed in R using the ‘alphashape3d’ package produced by Lafarge *et al.* (2014). The computation returns a Delaunay triangulation of the boundary points of the tree crown as defined by the α shape. This creates a surface of triangles with normal vectors. The normal vectors of these triangles provide the data needed for the computation of *S*_c_ as described in Fig. 3.

#### Sampling the vectors.

An α shape is computed for each tree crown using the points of the crowns that are inside the bounding box described above. This means that some parts of the α shape surface are positioned in the interior of the crown, and not relevant for the computation of surface complementarity. The relevant triangles of the α shape are selected by performing a two-way nearest-neighbour search on the triangle centre points (similar to an earlier step above). The selected triangle centre points correspond to the points *x* and *x′* in Fig. 3 and are used as input for eqns (2) and (3). The normal vectors of the selected triangles correspond to vectors *n*_*x*_ and *n*_*x*_′ of which the dot product is taken to compute the *S*_c_ value for the pair of tree crown surfaces.

### Statistics

Differences in surface complementarity between overlapping and non-overlapping pairs of trees were tested using an independent samples *t*-test. Relationships between crown surface complementarity and slenderness are tested using linear regression. All variables were tested for normality using the Shapiro–Wilk test (Shapiro and Wilk, 1965).

## RESULTS

### Pair-wise complementarity computations

**Fig. 6.**
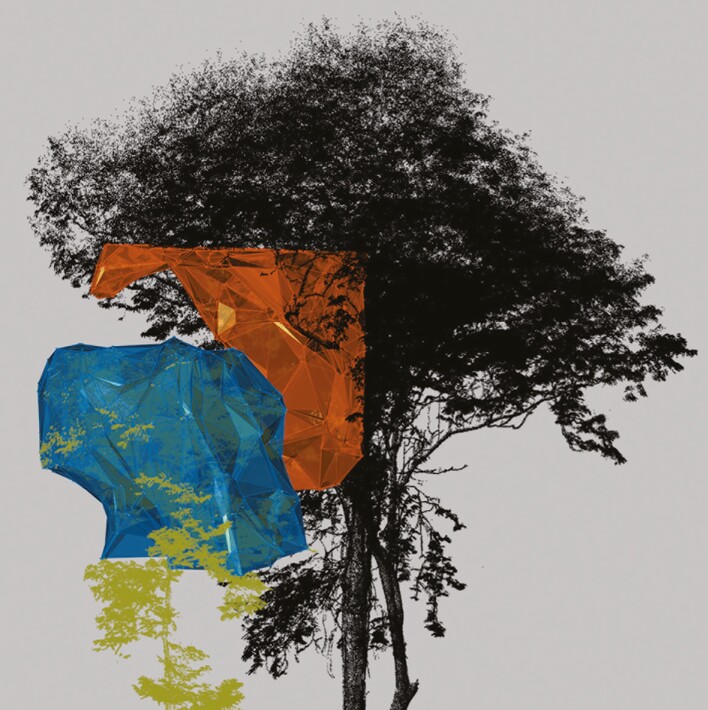
Convex and concave surfaces of a pair of tree crowns (pair 7, Table 2) modelled by the α shape. The coloured sycamore tree with a blue surface is tree 40_25 and the black tree with an orange surface is tree 80_22 (see Table 1).

**Fig. 7.**
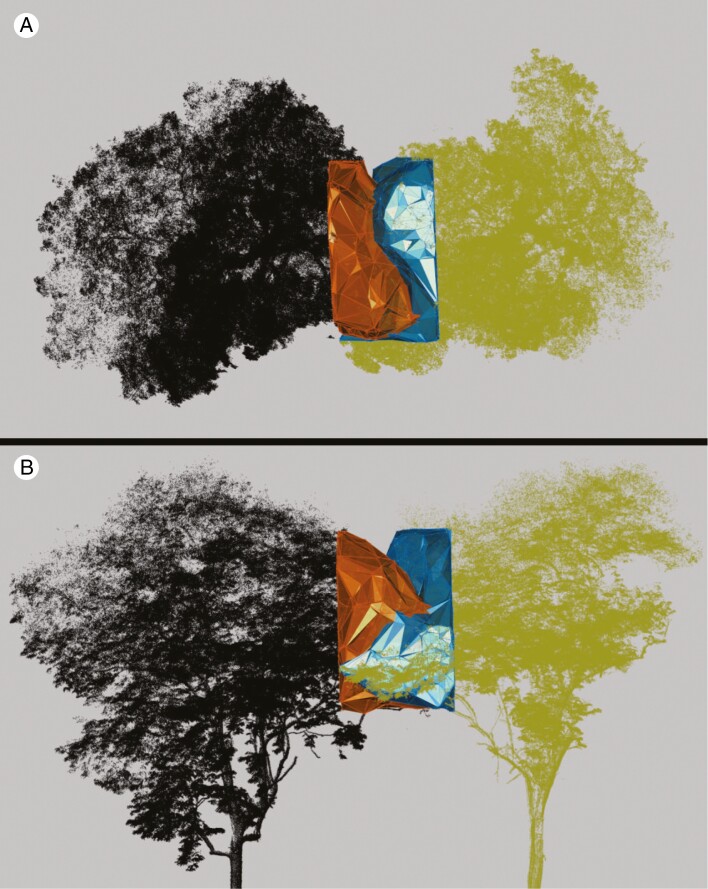
Top (A) and side (B) view of a pair of overlapping tree crowns (pair 3 in Table 2). The coloured sycamore tree with the blue α shape is tree 60_22 and the black tree with the orange α shape is tree 60_21.

Complementarity values were succesfully computed for all 14 pairs (Table 2). Half of the pairs showed no crown overlap. An example is shown in Fig. 6. The other half did show overlap between their crowns (Fig. 7).

**Fig. 8.**
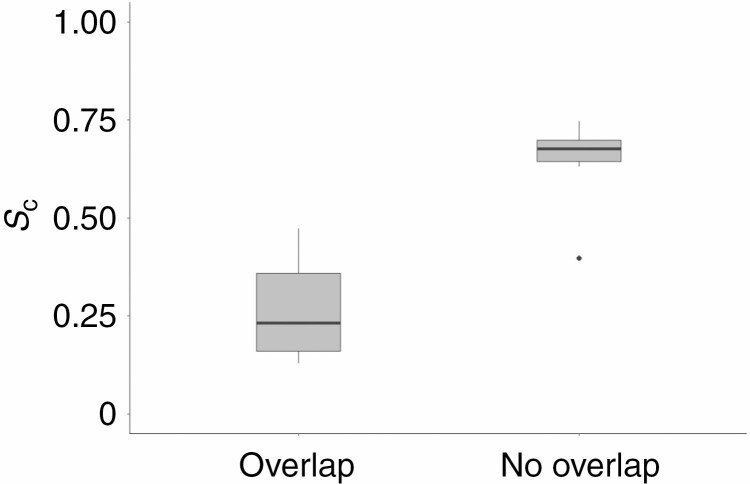
Boxplot of the complementarity values of overlapping and non-overlapping pairs of tree crowns. The box covers the range between the 25 and 75 % quartiles. Black dots represent outliers. Whiskers indicate minimum and maximum values (excluding outliers). Values were computed using a voxel size of 5 cm and α = 1. Mean *S*_c_ of overlapping pairs was significantly lower than that of non-overlapping pairs (significance level 0.05, *P* = 0.001).

The complementarity values of the overlapping pairs were significantly lower than those of non-overlapping pairs (Fig. 8, significance level 0.05, *P* = 0.001). The mean *S*_c_ value was 0.267 for overlapping crowns and 0.647 for non-overlapping crowns. The point clouds of the outlier pair (pair #11 in Table 2) in the non-overlapping group were of inferior quality since the point density in the interaction zone was much lower compared with other pairs. This may have resulted in an incorrect representation of the tree crown surfaces.

### Effect of α on complementarity measurements

**Fig. 9.**
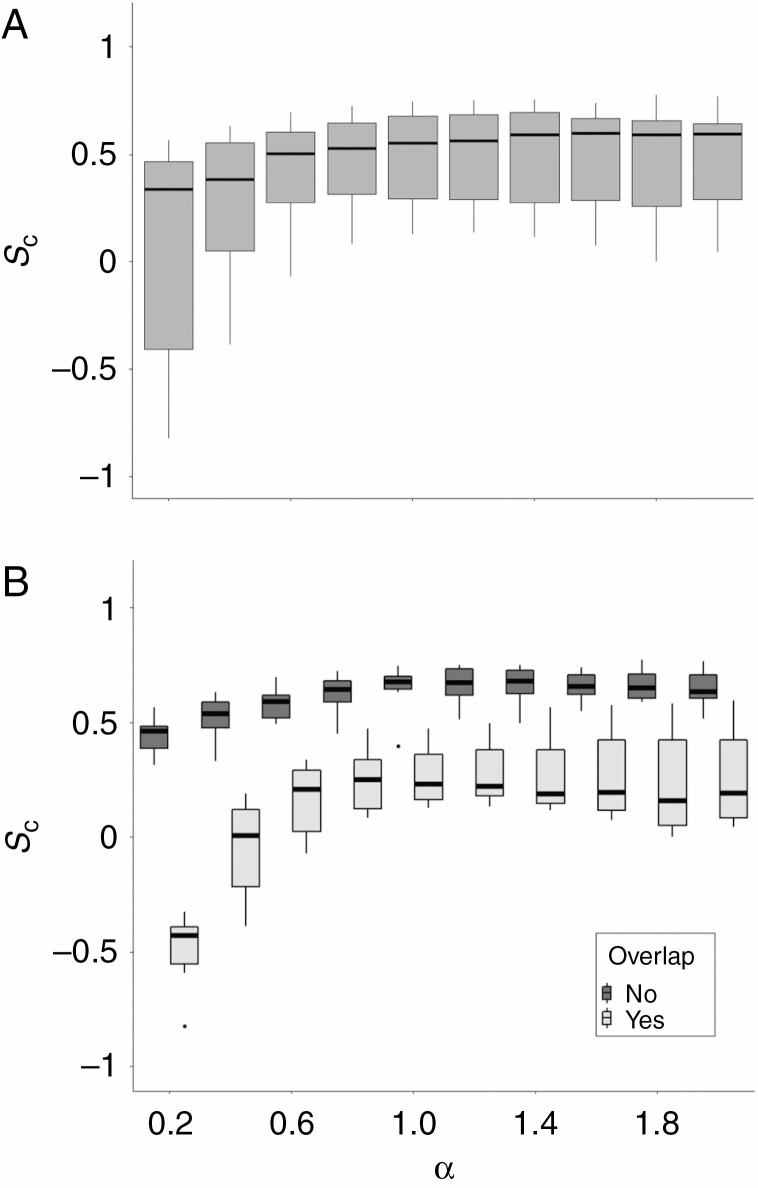
Boxplots of the average *S*_c_ value at different settings of α for all 14 pairs (A) and grouped into overlapping and non-overlapping pairs (B). In (B), the dark coloured boxplots are the non-overlapping pairs and the light coloured boxplots are overlapping pairs.

Sample mean *S*_c_ values were relatively low at small α values and increased with larger α values, stabilizing after α = 1 (Fig. 9A). The larger lower quantile and whisker of the boxplot at the lowest α value (α = 0.2) suggest that this effect is more pronounced in pairs with low complementary values. This is also observed in Fig. 9B, where the sample is split into overlapping and non-overlapping pairs. Sample mean *S*_c_ of overlapping pairs strongly increases over the range of α = 0.2 to α = 1, while non-overlapping pairs show only a minor increase. At values larger than α = 2.5, the mean *S*_c_ values of the sample decrease again (Fig. 11). The sample median was much less affected by this than the sample mean (Table 3).

**Table 3.** Sample mean and median surface complementarity values using a range of α values.

α	Mean *S*_c_	Median *S*_c_
0.2	0.05	0.36
0.4	0.28	0.41
0.6	0.39	0.43
1.0	0.48	0.55
2.0	0.48	0.59
3.0	0.43	0.56
5.0	0.32	0.43
10.0	0.23	0.29

### Slenderness and surface complementarity

Surface complementarity of a pair of tree crowns correlated positively with tree pair average slenderness (Fig. 11) but a substantial part of the variance in *S*_c_ remained unexplained (adjusted *R*^2^ = 0.484). In some pairs, the slenderness of the individual trees was similar (white dots close to each other) while other pairs showed large differences in slenderness (white dots far apart). The results suggest that a particular tree can grow a complementary crown with one neighbour, and not with another. For example, tree 80_22 (Table 1, row 15) is a large tree with a low slenderness coefficient. Paired with another non-slender neighbouring tree, the pair scores a low *S*_c_ value of 0.26 (Table 2, row 4, pair slenderness = 0.45). When paired with a slender neighbouring tree (Table 2, row 7, pair slenderness = 0.60), the pair scores a relatively high *S*_c_ value of 0.63.

## DISCUSSION

### Crown overlap and surface complementarity

A metric for surface complementarity, *S*_c_, was adopted from Lawrence and Colman (1993) and applied to point clouds of pairs of trees. This enabled the quantification of the puzzle-like pattern present in groups of tree crowns exhibiting crown shyness. The method produced sensible results, as overlapping crowns scored significantly lower in surface complementarity compared with non-overlapping crowns (Fig. 8). The surface complementarity values of non-overlapping crowns are similar to those that Lawrence and Colman found for the complexes of proteins they analysed, which ranged from 0.64 to 0.74. Since their models for molecules did not allow overlapping, they did not find such low values as for the overlapping crowns in this study.

Until recently, being able to observe crown shyness was reserved for structurally simple and flat canopies, where the backlighting of the sky reveals the gaps between tree crowns. The availability of point clouds reveals crown shyness throughout the vertical range of the canopy, suggesting that it might be more common than previously assumed. Hajek *et al.* (2015) already showed this by using terrestrial laser scanning-derived point clouds to measure the distances between neighbouring tree crowns along the contact zone of the crowns and relating these distances to signs of mechanical damage on terminal twigs. The present study expands existing methods for analysing crown shyness in 3-D by introducing a way to quantify the surface complementarity of tree crowns.

### The role of tree slenderness in crown shyness

For trees to be able to adapt their growth and avoid overlapping, they need to be aware of each other’s presence. Trees are sessile organisms, but wind can make them sway around, sometimes leading to crown collisions with adjacent trees. Slender trees sway more in the wind and are therefore more likely to collide with one another (Rudnicki *et al.*, 2008). Where other studies found that slender trees grow smaller crowns as a result of collisions (Meng *et al.*, 2006; Sharma *et al.*, 2017), our results suggest they also grow crown shapes that complement those of their neighbours. By doing so, trees optimize available growing space while minimizing damage from collisions.

The results of this study are in line with the theory that physical contact plays a role in the formation of crown shyness. The average slenderness of a pair of trees showed a positive relationship with the level of shape complementarity between the pair (Fig. 11). However, due to the limited number of samples, no concrete conclusions can be made based on this result. The low *P*-value (*P* = 0.003) indicates there may be an effect of tree pair slenderness on the complementarity of tree crowns, but it is not able to explain all the variation of crown surface complementarity in the sample by itself (adjusted *R*^2^ = 0.484). A possible reason for this is the influence of other factors that were not included in this analysis. For example, tree species may differ in susceptibility to damage from collisions (Hajek *et al.*, 2015). Two tree pairs of different species but similar slenderness may score different *S*_c_ as a result of this. Furthermore, light conditions were not considered in this study. Shaded tree crowns can show increased mortality of lateral branches and inhibited bud expansion which may strongly affect the development of a tree’s crown shape in the proximity of a neighbouring tree (Schoonmaker *et al.*, 2014).

### Choosing the α value

**Fig. 10.**
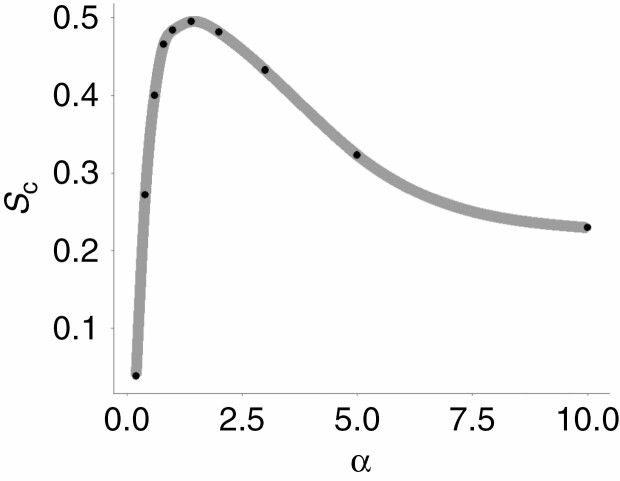
Average *S*_c_ of all 14 pairs for a range of α values (black dots). The grey line is a spline interpolation of the data points. Values are computed using a 5 cm voxel size.

The α value determines the level of detail of the α shape surface. The smaller the value, the smaller the cavities in the surface are. Higher α values increase the minimum size of the cavities, leading to a more convex surface (Edelsbrunner and Mucke, 1994). The selected α value affected the computation result in several ways. The sample mean surface complementarity value increased considerably over the α range from 0 to 1 (Figs 9 and 10). This effect was strong for the sample mean but not for the sample median (Table 3), indicating that pairs scoring high complementarity values were less affected by this than the overlapping pairs scoring low complementarity values.

**Fig. 11.**
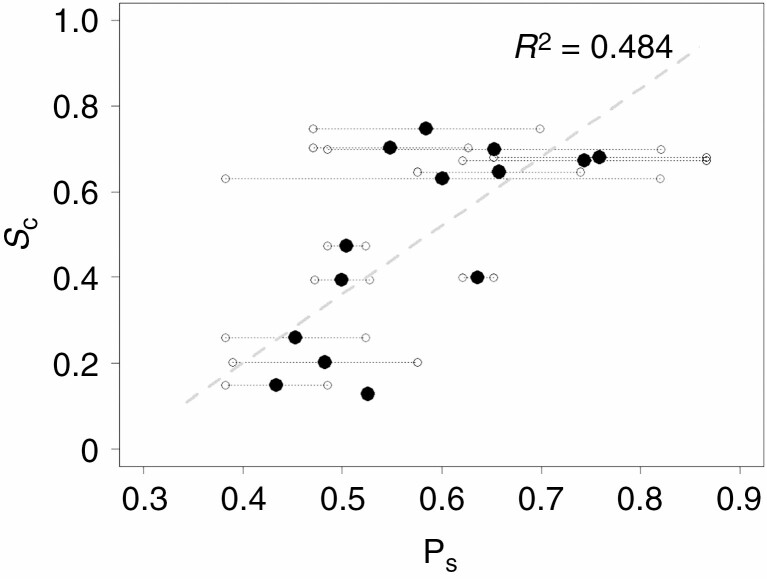
Linear regression of crown surface complementarity (*S*_c_) and average slenderness of a pair of trees (black dots). Small circles connected to black dots indicate the slenderness of the individual trees in a pair. Crown surface complementarity showed a significant positive correlation with pair slenderness (*P* = 0.003 and adjusted *R*^2^ = 0.484) at the 0.05 significance level. *S*_c_ values were calculated using α = 1.

Overlapping sections of surfaces occur at the extremities of the tree crowns, i.e. the branches. When the α value decreases, the positions of the surface triangles at the tips of the branches barely change because there is no space left to carve out by the shrinking α sphere). In non-overlapping sections on the other hand, surfaces generated with a large α value contain triangles that will move towards their respective trees to accommodate the space being carved out between the branches by the shrinking α sphere. This effect creates distance between triangles on non-overlapping sections of the tree crown surfaces, while triangles at the ends of branches stay close to each other. As a consequence, the triangles on non-overlapping sections are less likely to be selected by the nearest-neighbour search between triangle centre points that is performed to select the normal vectors for computing *S*_c_. Since non-overlapping sections produce positive surface normal vector dot products (Fig. 4), it is expected that *S*_c_ values will decrease as their contribution to the surface normal vector sample reduces compared with that of the overlapping sections at branch tips.

In the range 1 < α < 2, the computations produced stable results in terms of *S*_c_ sample distributions (Fig. 9). The decrease in mean surface complementarity of the sample when using α values >2 can be attributed to the loss of concave features of the tree crowns as the α shapes approach the convex hull of the tree point clouds. The increased convexity of the α shapes leads to lower surface complementarity values through a higher likelihood of overlapping and the conjunction of convex surface parts (Fig. 4A, B). Using α shapes computed with 1 < α < 2, it was possible to adequately capture both convex and concave features of the tree crown surfaces.

### Limitations

A limitation of this study was the availability of point clouds of adjacent trees. This resulted from the conscious choice of isolated trees in the original study (Lau *et al.*, 2019). A larger sample size would provide a more reliable basis for statistical inference, and this is planned for further studies.

Related to this issue is the problem of scalability. For a sample of 14 trees it was viable to manually check for segmentation errors, but for larger numbers of samples this procedure becomes increasingly laborious. Many efforts are being made to improve individual tree segmentation methods (Itakura and Hosoi, 2018; Yan *et al.*, 2018; Williams *et al.*, 2019); however, distinguishing tree crowns, especially in structurally complex forests, remains one of the main challenges (Burt *et al.*, 2019).

When applying the method presented in this study to other point clouds, it is important to consider the point density in the contact zone. When using a terrestrial laser scanner, occlusion can lead to low point densities in the upper regions of the canopy (Wilkes *et al.*, 2017). One pair that was used in this study suffered from a low point density, characterized by empty space and a lack of clearly defined branches in the contact zone. This led to the surfaces of both tree crowns being strongly concave, possibly resulting in an underestimation of the surface complementarity of the pair (outlier in Fig. 8). The effect of false surface concavity due to low point density could be compensated by using a larger α value. However, to keep samples comparable, this larger α value would also need to be applied to pairs with sufficient point density. As shown in Fig. 10, large α values reduce the possibility to detect surface complementarity due to loss of concave features. Therefore, to guarantee a reliable estimate of crown surface complementarity, it is recommended to use point clouds which have a sufficient point density to accurately capture the structure of the tree crowns in the contact zone.

### CONCLUSIONS

Crown shyness creates an impressive puzzle-like structure of complementary tree crowns in forest canopies. The acquisition of detailed 3-D tree representations from terrestrial laser scanning allows the quantification of this remarkable characteristic of trees displaying crown shyness. We measured the crown complementarity of pairs of trees and found that pairs of slender trees grow more complementary crowns, suggesting that adjacent trees may adapt the shape of their crown as a result of physical touch. This study serves as an example of the value of 3-D tree modelling for expanding our understanding of canopy interactions as it helped both visualizing and quantifying an interesting canopy dynamic in unprecedented ways.
